# Sociodemographic factors associated with trajectories of depression among urban refugee youth in Kampala, Uganda: A longitudinal cohort study

**DOI:** 10.1017/gmh.2024.135

**Published:** 2024-12-16

**Authors:** Zerihun Admassu, Sikky Shiqi Chen, Carmen H. Logie, Moses Okumu, Frannie MacKenzie, Robert Hakiza, Daniel Kibuuka Musoke, Brenda Katisi, Aidah Nakitende, Peter Kyambadde, Lawrence Mbuagbaw

**Affiliations:** 1 Factor-Inwentash Faculty of Social Work, University of Toronto, Toronto, ON, Canada; 2 United Nations University Institute for Water, Environment, and Health, Hamilton, ON, Canada; 3 Centre for Gender & Sexual Health Equity, Vancouver, BC, Canada; 4 Women’s College Research Institute, Women’s College Hospital, Toronto, ON, Canada; 5 School of Social Work, University of North Carolina Chapel Hill, Chapel Hill, NC, USA; 6 School of Social Sciences, Uganda Christian University, Mukono, Uganda; 7 Young African Refugees for Integral Development (YARID), Kampala, Uganda; 8 International Research Consortium (IRC-Kampala), Kampala, Uganda; 9 Most At Risk Population Initiative Clinic, Mulago Hospital, Kampala, Uganda; 10 National AIDS Control Program, Ministry of Health, Kampala, Uganda; 11 Department of Health Research Methods, Evidence and Impact, McMaster University, Hamilton, ON, Canada; 12 Biostatistics Unit, Father Sean O’Sullivan Research Centre, St Joseph’s Healthcare, Hamilton, ON, Canada; 13 Centre for Development of Best Practices in Health (CDBPH), Yaoundé Central Hospital, Yaoundé, Cameroon; 14 Division of Epidemiology and Biostatistics, Department of Global Health, Stellenbosch University, Cape Town, South Africa

**Keywords:** depression, refugee, trajectories, youth

## Abstract

**Background:**

There is a high prevalence of depression among refugee youth in low- and middle-income countries, yet depression trajectories are understudied. This study examined depression trajectories, and factors associated with trajectories, among urban refugee youth in Kampala, Uganda.

**Methods:**

We conducted a longitudinal cohort study with refugee youth aged 16–24 in Kampala, Uganda. We assessed depression using the Patient Health Questionnaire-9 and conducted latent class growth analysis (LCGA) to identify depression trajectories. Sociodemographic and socioecological factors were examined as predictors of trajectory clusters using multivariable logistic regression.

**Results:**

Data were collected from n = 164 participants (n = 89 cisgender women, n = 73 cisgender men, n = 2 transgender persons; mean age: 19.9, standard deviation: 2.5 at seven timepoints; n = 1,116 observations). Two distinct trajectory clusters were identified: “sustained low depression level” (n = 803, 71.9%) and “sustained high depression level” (n = 313, 28.1%). Sociodemographic (older age, gender [cisgender women vs. cisgender men], longer time in Uganda), and socioecological (structural: unemployment, food insecurity; interpersonal: parenthood, recent intimate partner violence) factors were significantly associated with the sustained high trajectory of depression.

**Conclusions:**

The chronicity of depression highlights the critical need for early depression screening with urban refugee youth in Kampala. Addressing multilevel depression drivers prompts age and gender-tailored strategies and considering social determinants of health.

## Impact statement

This study provides unique insights into the prevalence and persistence of depression among urban refugee youth in Kampala, Uganda. To our knowledge, this longitudinal study is unique in investigating the trajectories of depression, and factors associated with these trajectories, among urban refugees in Kampala. Our findings reveal that while some youth consistently experience heightened levels of depression, others maintain comparatively lower levels. Further, we identify sociodemographic (gender, age); structural (unemployment, food insecurity); and interpersonal (parenthood, recent intimate partner violence [IPV]) level factors that shape these distinct trajectories of depressive symptoms among refugee youth. Understanding these factors is essential for developing targeted interventions. Tailored mental health services and supportive resources are urgently needed for those experiencing persistent depression, addressing the unique stressors and traumas associated with displacement. Insights into protective factors and resilience mechanisms among refugee youth with lower depression levels can inform preventive strategies to enhance mental well-being across broader populations. By emphasizing these divergent experiences, our study emphasizes the necessity for nuanced mental health policies and programs that accommodate the diverse psychological responses and mental health needs of young refugees. Implementing such approaches can significantly enhance the quality of life and improve long-term outcomes for young refugees in urban, low-income settings like Kampala.

## Introduction

Mental health challenges are the leading contributors to the global burden of disease (Arias et al., 2022). Among these, depression is an increasing concern for adolescents (Shorey et al., 2022; Lu et al., 2024), especially for those from lower socioeconomic statuses. Despite the general scarcity of epidemiological data in low- and middle-income countries (LMICs), prevalence rates of up to 28% for significant depressive symptoms were noted among youth from 17 LMICs, including Uganda (Yatham et al., 2018). Forcible displacement is associated with elevated risks of adolescent depression (Thapar et al., 2022). A recent systematic review of 25 studies indicated a depression rate of 33% in child and adolescent refugee populations, including those living in Sub-Saharan Africa (Jin et al., 2021). Urgent attention is warranted to better understand, and address, drivers of depression among young refugees, particularly in LMICs where there is a scarcity of mental health resources (Lora et al., 2017).

Uganda is Africa’s largest refugee-hosting nation with over 1.5 million refugees in 2023 (United Nations High Commissioner for Refugees [UNHCR], 2021; 2023) and its urban city, Kampala, hosts over 93,000 refugees, of whom more than a quarter (26%) are youth aged 15–24 (UNHCR and Government of Uganda, 2022). Many of Kampala’s refugees live in informal settlement and slum contexts, and these settings may elevate vulnerability to mental health challenges due to social environments that include stressors, such as poverty, water insecurity, inadequate social support, restricted access to healthcare and limited opportunities of employment (Kamara et al., 2019; Alaazi and Aganah, 2020). A prior cross-sectional study reported a depression prevalence of approximately 28% among urban refugee youth living in informal settlements in Kampala (Logie et al., 2022).

The social–ecological framework is particularly relevant for investigating depression as it considers the interplay between social and ecological systems, spanning multilevel contexts, such as structural and institutional (e.g., systems shaping access to resources), community (e.g., social support), interpersonal (e.g., relationship dynamics) and intrapersonal (e.g., individual attitudes) domains (McLeroy et al., 1988). Among urban refugee youth in Kampala, associations have been found between increased adolescent depression and factors at the structural (e.g., food insecurity) and interpersonal (e.g., experiencing violence) levels (Logie et al., 2022). However, there is limited literature regarding the associations of socioecological factors and depression trajectories in Uganda and LMIC.

Within the existing literature, researchers have identified associations between depression trajectories and socioecological factors on the structural and interpersonal levels in non-refugee populations in high-income settings. For instance, at the structural level, food insecurity was associated with high-risk trajectories of depression, as evidenced by data collected from North American adolescents (McIntyre et al., 2017; Hatem et al., 2020). At the interpersonal level, longitudinal studies conducted in British and Dutch populations (Meijer et al., 2019; Weavers et al., 2021) found that adverse childhood experiences, particularly early exposure to violence, were associated with a heightened risk of persistent depression during adolescence. In addition, sociodemographic factors may contribute to variations in the course and persistence of depression. For instance, a systematic review examining depressive symptoms among adolescents across European and American countries has identified gender (cisgender women vs. cisgender men) and low socioeconomic status as sociodemographic predictors of high or increasing depression trajectories (Shore et al., 2018).

Differences in depression among refugee adolescents are shaped by sociodemographic characteristics. Cross-sectional studies conducted among young refugee from LMICs, such as Uganda and Syria, have revealed the significant relationships between depression and various sociodemographic factors, including age, gender, length of time in displacement, household background and education (Scherer et al., 2020; Logie et al., 2022).

Few trajectory analyses on mental health of young refugees have been conducted, and these appear to primarily focus on unaccompanied refugee minors resettled in European high-income countries (HICs). For example, a study involving asylum-seeking adolescents in Germany found four depression trajectories (i.e., unremarkable, reacting, adapted and persisting) during their first-year of resettlement, highlighting the association between changes in asylum status and decreasing depressive symptoms (Muller et al., 2019). Similarly, research conducted in Sweden on long-term antidepressant use among refugee youth revealed four trajectory groups: low constant, low increasing, medium decreasing and high increasing, with the duration of stay in Sweden and comorbid mental disorders identified as the most significant factors distinguishing the trajectories (Rahman et al., 2022).

Although there is an evidence-base indicating high prevalence of depression among displaced populations (Bedaso and Duko, 2022), including refugee youth in Uganda (Kaggwa et al., 2022), there is a dearth of studies on trajectories of depression in Uganda – and LMIC at large – with young refugees. Longitudinal investigations could capture temporal changes and identify developmental patterns in depressive symptoms, advancing knowledge beyond cross-sectional studies in terms of individual trajectories, differences in pathways and variability in depression outcomes over time among urban refugee youth. Better understanding factors associated with the patterning of depressive symptoms among young refugees in Uganda can inform tailored prevention and treatment services. To address this knowledge gap, this study 1) explored trajectories of depression patterns among urban refugee youth in Kampala, Uganda and 2) examined sociodemographic and socioecological predictors of depression trajectories.

## Methods

### Participants

Study participants were recruited from five informal settlements in Kampala, Uganda (Kabalagala, Kansanga, Katwe, Nsambya and Rubaga) (Logie et al., 2021), using purposive sampling methods, such as word-of-mouth and site-based sampling at the Young African Refugees for Integral Development center, a youth-focused community-based nongovernmental organization. Eligibility criteria included: 1) current residence in one of the five selected informal settlements in Kampala; 2) identification as a displaced person, refugee or having a parent who is a refugee or displaced; 3) age between 16 and 25 years; 4) ownership of or daily access to a mobile phone and 5) fluency in French, English, Kirundi, Kinyarwanda or Swahili.

### Study procedures

Data for this study were derived from a longitudinal cohort study involving urban refugee youth, with seven waves of survey data collected at 6-month intervals from 2020 to 2024. Recruitment and data collection were facilitated by trained research assistants and 12 peer navigators (six young women and six young men) aged 16–24, all residing in one of the five informal settlements. The peer navigators administered standardized questionnaires using a secure, tablet-based application called SurveyCTO (Dobility, Cambridge, USA). To ensure diverse representation, the questionnaires were made available in French, English, Kirundi, Kinyarwanda and Swahili, allowing participants to select their preferred language.

Prior to the actual survey, the study tool was piloted in the same setting with participants similar to the main study population. This piloting process ensured the tool’s relevance, cultural appropriateness and clarity for the target population. To maintain confidentiality, each participant was assigned a unique case ID, and no personally identifying information was stored in the research dataset.

### Measurements

The primary study outcome was depression, assessed using the Patient Health Questionnaire-9 (PHQ-9), demonstrating good internal consistency (Cronbach’s alpha = 0.82). The total scores for the nine items in the PHQ-9 were summed, resulting in a composite score ranging from 0 to 27, where higher scores indicate higher levels of depression severity (Kroenke and Spitzer, 2002). The PHQ-9 has been validated for assessing depression in Uganda (Nakku et al., 2016) (Logie et al. 2020b).

Aligned with social–ecological framework (McLeroy et al., 1988), predictor variables included baseline sociodemographic characteristics (age, gender, country of birth and years living in Uganda); *interpersonal-level* factors (parenthood status, recent [past 3-month] IPV) and *structural-level* factors (education, employment and food insecurity).

#### Interpersonal level

We assessed if participants had children as a binary variable (yes/no). Recent IPV was measured using the revised Conflict Tactics Scale (Straus and Douglas, 2004a), which includes three subscales: psychological (three items), sexual (two items) and physical (three items) violence experiences. Each of these subscale variables was separately coded as ‘yes’ for participants who experienced violence and ‘no’ for those who did not experience any form of IPV in the past 3 months.

#### Structural level

We assessed the highest level of education (less than secondary, some secondary and secondary or higher) and employment (student, employed and unemployed) as a categorical variables. Food insecurity was measured using a single-item question: “How often do you go to bed hungry because you didn’t have enough to eat?” with four response options: Never, Sometimes, Most days and Everyday.

### Ethical considerations and approvals

Ethical approvals were obtained from University of Toronto Research Ethics Board (REB) (reference 37496), the Mildmay Uganda Research Ethics Committee (0806-2019) and the Uganda National Council for Science & Technology (reference HS 2716). All participants provided written informed consent, affirming their voluntary participation and understanding of the study’s objectives and procedures.

### Statistical analysis

We used latent class growth analysis (LCGA) to specify the distinct trajectory patterns of depression scores among youth refugees. LCGA assumes that the heterogeneity in a single outcome repeatedly assessed can be summarized by a certain number of clusters with distinct cluster-specific trajectories (Herle et al., 2020). Considering the small study sample size, LCGA models with one to three classes were sequentially fitted, starting with a one-cluster model (whereby it assumes that all individuals had the same trajectory) and then adding another cluster for each successive model. For each model, allocation of individuals was based on maximum posterior probability (Jung and Wickrama, 2007).

In determining the ideal number of classes that best fit the data, several goodness-of-fit criteria were evaluated (Nylund et al., 2007). These included the Akaike information criteria, the Bayesian information criteria (BIC) and the sample-size adjusted BIC, with lower scores indicate better fitting models (Jung and Wickrama 2008). Additionally, factors, such as the size of the smallest class (>5%), higher entropy and the interpretability of latent trajectories were taken into account during class selection. Furthermore, alongside the goodness-of-fit indices, the final determination of the optimal number of clusters relied on examining averaged group membership posterior probabilities and ensuring the distinctiveness of each class.

Following the results of the LCGA analysis, chi-square tests and one-way univariate analyses of variance were executed to examine the potential variations in social–ecological characteristics among different trajectory clusters. Logistic regression analyses were also conducted to predict class-membership using baseline factors, where class-membership treated as outcome variable. All analyses were set at a level of significance of 0.05. The LCGA analysis was carried out in Mplus Version 8.0 using the robust maximum likelihood estimator. Full information maximum likelihood was used to manage missing data), while regression analyses were performed using StataSE 18.0 (Stata Corp LP, College Station, TX).

## Results

### Participant characteristics

The study initially included n = 164 participants at baseline (Wave 1). For the LCGA, we used the continuous depressive symptom scores from all available data points across the seven waves of data collection. The number of participants with data at each wave varied slightly due to missing observations: n = 148 at Wave 2, n = 162 at Wave 3, n = 162 at Wave 4, n = 161 at Wave 5, n = 159 at Wave 6 and n = 161 at Wave 7. This resulted in a total of 1,116 observations used in the LCGA model to identify latent classes of depressive symptom trajectories.

Participants’ ages ranged from 16 to 24 years, with an average age of 19.9 years (standard deviation [SD] = 2.5). More than half of the participants identified as cisgender women (n = 89, 54.3%), followed by cisgender men (n = 73, 44.5%) and two individuals (1.2%) identified as transgender. Table 1 provides a summary of participants’ characteristics.

**Table 1.** Sociodemographic and socioecological characteristics among refugee youth participants, Kampala, Uganda (N = 164)

Variables	Mean (SD)/N (%)
*Sociodemographic factors*	
Age	19.9 (2.5)
Gender	
Cisgender women	89 (54.3%)
Cisgender men	73 (44.5%)
Transgender	2 (1.2%)
Birth country	
DRC	112 (68.3%)
Burundi	25 (15.2%)
Sudan/South Sudan	9 (5.5%)
Other	18 (11.0%)
Years living in Uganda	
<1 year	7 (4.3%)
1–5 years	84 (51.2%)
6–10 years	50 (30.5%)
>10 years	23 (14.0%)
*Interpersonal factors*	
Having children	
No	151 (92.1%)
Yes	13 (7.9%)
Psychological violence	
No	144 (88.3%)
Yes	20 (11.7%)
Sexual violence	
No	154 (94.5%)
Yes	10 (5.5%)
Physical violence	
No	152 (93.2%)
Yes	12 (6.8%)
*Structural factors*	
Education n = 2	37 (22.8%)
Less than primary	65 (40.1%)
Some secondary	60 (37.1%)
Secondary+	
Employment n = 3	58 (36.0%)
Unemployed	59 (36.6%)
Student	44 (27.4%)
Employed (paid/unpaid)	
Food insecurity frequency n = 1	
Never	54 (33.1%)
Sometimes	101 (62.0%)
Most days	6 (3.7%)
Everyday	2 (1.2%)

*Note:* n = missing case; SD = standard deviation; N = total number; DRC= Democratic Republic of the Congo

### Trajectory clusters of depressive symptoms among youth refugees

The analysis, considering goodness-of-fit criteria, cluster distinctiveness and the alignment of participants with their assigned clusters, revealed that the two-cluster solution best captured the trajectory of depression symptoms (Table 2).

**Table 2.** Fit indices of latent class growth analysis of depression trajectories: two- to three-class solutions

Number of classes	AIC	BIC	aBIC	Entropy	Trajectory Group Prevalence (%)		
					1	2	3
2-class	6,784.629	6,829.786	6,801.200	0.804	71.9	28.1	
3-class	6,850.197	6,935.646	6,881.649	0.732	28.8	21.6	49.6

*Note:* AIC, Akaike information criterion; BIC, Bayesian information criterion, aBIC, adjusted Bayesian information criterion.

**Figure 1.**
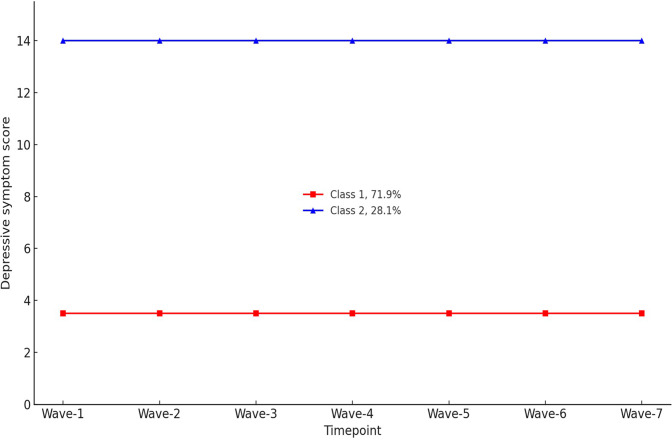
Trajectory classes of depressive symptoms based on participants’ PHQ-9 scores (N = 1,116).

Class 1 (labeled as “sustained low depression level”; n = 803, 71.9%) consistently maintained a low and stable level of depression scores across time, with PHQ scores ranging from 3.0 to 4.0, which fall within the ‘none’ to minimal depression severity category (Kroenke et al., 2001). Class-2 (labeled as “sustained high depression level”; n = 313, 28.1%) exhibited persistently high depressive symptom scores, remaining stable over time and ranging from 13.0 to 14.0, indicating moderate depression severity. According to Kroenke et al. (2001), a PHQ-9 score ≥10 has a sensitivity of 88% and a specificity of 88% for major depression. Both classes revealed high average posterior probabilities for their assigned class, with average posterior probabilities of the most likely class membership at 0.961 and 0.906 for classes 1 and 2, respectively, indicating that the majority of individuals were clearly assigned to a specific cluster (see Figure 1).

### Determinants for trajectory clusters of depressive symptoms

Univariate analyses revealed significant associations between trajectories of depressive symptoms and baseline factors including age, length of stay in Uganda, parenthood status, employment status, experience of IPV and food security status.

In a multivariate logistic regression analyses adjusting for age and gender, several factors were identified to be significantly associated with sustained high depression trajectory. Sociodemographic factors included older age (adjusted only for gender; adjusted odd ratio [aOR] = 1.13; 95% confidence interval [CI]: 1.06, 1.19; P < 0.001), gender (cisgender women vs. cisgender men) (adjusted only for age; aOR = 1.45; 95% CI: 1.10, 1.92; P = 0.009) and longer duration of stay in Uganda (more than 10 years vs. less than 1 year; aOR = 2.87; 95% CI: 1.20, 6.86; P = 0.018).

In addition, significant associations were also revealed between a sustained high depression trajectory and different participant characteristics. These included interpersonal level factors, such as having dependents (aOR = 2.28; 95% CI: 1.40, 3.70; P = 0.001), experiencing recent sexual violence (aOR = 2.89; 95% CI: 1.68, 4.96; P < 0.001) and recent physical violence (aOR = 2.70; 95% CI: 1.66, 4.40; P < 0.001), and structural level factors including current unemployment (vs. currently employed; aOR = 2.91; 95% CI: 2.04, 4.15; P < 0.001), and food insecurity (everyday vs. never; aOR = 9.76; 95% CI: 2.59, 36.70; P = 0.001). The factors associated with trajectories of depressive symptoms are displayed in Table 3.

**Table 3.** Associations between baseline sociodemographic and socioecological characteristics and depression trajectories among refugee youth in Kampala, Uganda

Variables	Trajectory: consistently high depressive symptoms (Ref. sustained low depressive symptoms)			
	Unadjusted OR (95% CI)	P-value	Adjusted OR (95% CI)	P-value
*Sociodemographic factors*				
Age	1.11 (1.06, 1.18)	<0.001	1.13 (1.06, 1.19)	<0.001
Gender				
Cisgender women (*vs* men)	1.27 (0.97, 1.66)	0.079	1.45 (1.10, 1.92)	0.009
Years living in Uganda (Ref. < 1 year)	1.65 (0.72, 3.76)	0.236	1.59 (0.69, 3.65)	0.271
1–5 years	3.42 (1.49, 7.87)	0.004	3.08 (1.33, 7.10)	0.008
6–10 years	3.22 (1.35, 7.64)	0.008	2.87 (1.20, 6.86)	0.018
> 10 years				
Birth country (Ref. DRC)				
Burundi	0.99 (0.69, 1.43)	0.975	0.97 (0.66, 1.42)	0.874
Sudan/South Sudan	0.21 (0.08, 0.54)	0.001	0.22 (0.09, 0.56)	0.001
Other	1.09 (0.72, 1.65)	0.672	1.16 (0.76, 1.76)	0.490
*Interpersonal factors*				
Having children (Yes vs. No)	2.97 (1.91, 4.61)	<0.001	2.28 (1.40, 3.70)	0.001
Experiences of IPV (past 3-month)				
Psychological violence (Yes vs. No)	1.72 (1.17, 2.51)	0.005	1.44 (0.96, 2.13)	0.074
Sexual violence (Yes vs. No)	3.62 (2.15, 6.09)	<0.001	2.89 (1.68, 4.96)	<0.001
Physical violence (Yes vs. No)	3.22 (2.00, 5.16)	<0.001	2.70 (1.66, 4.40)	<0.001
*Structural factors*				
Education (Ref. less than primary)				
Some secondary	1.33 (0.94, 1.90)	0.110	1.09 (0.75, 1.57)	0.650
Above secondary level	1.13 (0.79, 1.63)	0.498	0.81 (0.55, 1.20)	0.289
Employment (Ref. employed [paid/unpaid])				
Student	1.05 (0.73, 1.51)	0.786	1.36 (0.92, 2.01)	0.125
Unemployed	2.60 (1.84, 3.66)	<0.001	2.91 (2.04, 4.15)	<0.001
Food insecurity frequency (Ref. never)				
Sometimes	0.95 (0.72,1.26)	0.729	0.86 (0.65, 1.15)	0.326
Most days	0.90 (0.44, 1.86)	0.777	0.92 (0.44, 1.93)	0.837
Everyday	8.46 (2.28, 31.36)	0.001	9.76 (2.59, 36.70)	0.001

*Note:* OR, odds ratio; CI, confidence interval; IPV, intimate partner violence; Ref, reference group(s); adjusted OR, adjusted for age and gender. Transgender participants were excluded from regression analyses due to the small sample size.

## Discussion

The results of this longitudinal study demonstrated notable stability and chronicity of depression among urban refugee youth in Kampala. Two distinct trajectory groups were identified: one reflected sustained low levels of depression among over one-quarter of participants, while the other identified sustained high levels of depression. We also found sociodemographic characteristics (i.e., older age, gender [women vs. men]; longer duration of stay in Uganda); structural level factors (unemployment, food insecurity) and interpersonal level factors (parenthood and recent IPV experiences) that significantly differentiated the trajectories of depressive symptoms among this sample refugee youth. These findings offer new insight into the persistence and drivers of depression among urban refugee youth and directions for intervention.

Our study identified widespread chronic depression with this sample of urban refugee youth, whereby 28.1% of our sample demonstrated persistently high levels of depressive symptoms across seven study waves. Their PHQ-9 scores (13.0–14.0) reflect a moderate severity of depression (Kroenke et al., 2001). These findings are consistent with prior research among young refugees in Uganda that documented depression prevalence of 24–33% (Kaggwa et al., 2022), including among urban refugee youth in Kampala, whereby 27–29% reported moderate–severe symptoms of depression (Logie et al., 2022). Most of this sample, however, reported a sustained low depression trajectory and were in the category of none-minimal depression based on the PHQ-9 scores (3.0–4.0), echoing the findings of non-refugee youth populations across a range of American and European countries that the majority presented ‘no or low’ depressive symptom trajectories (Shore et al., 2018). Our study identified a constant and relatively unchanged trajectory of high depressive symptoms, aligning with other longitudinal studies with unaccompanied refugee minors who had resettled in Europe (Vervliet et al., 2014; Jakobsen et al., 2017). The stable pattern of high-symptom depression is hypothesized to result from trauma and concurrent social–ecological stressors. While our findings contrasted with the fluctuating patterns of depression identified in general adolescent populations in North American HICs (Yaroslavsky et al., 2013; Ellis et al., 2017) and conflict-affected young people in LMICs (Purgato et al., 2020), this may be due to differences in sample characteristics, such as the stage of adolescence (mid-adolescence vs. late adolescence), the prevalence of substance use and levels of social support.

The results of our study corroborate and build upon prior research on sociodemographic factors associated with depression trajectories among urban refugee youth. Specifically, we found that a longer duration of stay in Uganda was linked with a stable and higher depressive symptom trajectory. Our findings corroborate studies conducted in refugee camps in LMICs, which have suggested a deterioration in mental health among refugees with an increased length of stay (Braun-Lewensohn and Al-Sayed, 2018; Getnet et al., 2019). Previous findings indicated that as refugee youth spent more time in settlements, rather than experiencing increased adaptation and acculturation, they tended to demonstrate lower resilience and greater hopelessness (Braun-Lewensohn and Al-Sayed, 2018). This phenomenon could be attributed to the absence of social support and ongoing contexts of adversity, with cumulative and daily stressors including poverty, unemployment, language barriers, discrimination and resource scarcity (Afifi et al., 2016; Silove et al., 2017; Akgul et al., 2019).

In terms of interpersonal level socioecological factors, our study found recent experiences of IPV as significant predictors of the sustained trajectory of high depressive symptoms. This finding corresponded to several studies involving refugee youth in Uganda, which have pinpointed the association between violence exposure and greater depression in both camp settlements (Meyer et al., 2017a, 2017b) and urban contexts (Logie et al., 2020a). The long-term impacts of violence exposure have been detailed in previous studies, especially regarding disrupted neural system development and stress response pathways, which could result in emotional dysregulation and chronic adverse health effects (McLaughlin et al., 2014; Slavich and Irwin, 2014). In addition, existing evidence has demonstrated gender and age-based disparities in influences of violence experiences on refugee youth’s mental health, including that younger refugee women with recent experiences of IPV had elevated risks of depression, anxiety and post-traumatic stress disorder (Hossain et al., 2021; El-Moslemany et al., 2022). Considering the distinct dynamics of IPV experiences among refugee men and women, future research should further investigate the role of gender in the relationship between IPV and depression trajectories through gender-disaggregated analyses.

Besides, we also found that parenting refugee youth showed a greater likelihood of high depressive symptom trajectory compared to those who were non-parenting. This finding was consistent with prior studies with refugee young women in Kampala (Logie et al., 2019). Future research should further explore the community level factors that would contribute to chronic depression among refugee youth of parenthood, such as stigma and lower social support, disrupted education, entrenched cycles of poverty and stigma (Steventon Roberts et al., 2022).

Our findings indicated that food insecurity was a significant structural-level determinant of chronic depression among urban refugee youth. Food insecurity disproportionately affects refugee populations in LMICs (Maharaj et al., 2017) and detrimental impacts on mental health outcomes among young refugees from LMICs, including anxiety, depression and post-traumatic stress disorder, are well-established across various contexts (Elgar et al., 2021; Pakravan-Charvadeh et al., 2021; Abou-Rizk et al., 2022). The high depressive symptom trajectory observed among refugee adolescents struggling with insufficient food provision may be explained by persistent feelings of deprivation due to chronic poverty (Emerson et al., 2017). The decrease in food availability and dietary quality may often coincide with refugees’ resettlement in camps and host countries, where they may encounter challenges such as difficulties in access to transport, unfamiliar food choices, limited affordable quality foods and a lack of nutrition knowledge (Emerson et al., 2017; Maharaj et al., 2017). Adoption of food-coping strategies to manage food shortages (e.g., reducing the number of meals, choosing fewer kinds of food groups and relying on less preferred or less expensive foods) and limited dietary diversity may exacerbate this burden of food insecurity, especially for women who often bear greater responsibilities for feeding and reproduction (Napier et al., 2018; Food and Agriculture Organization, 2021). Therefore, future research may benefit from further examining the roles of gender norms, values and expectations in the associations between food insecurity and depression trajectories.

## Strengths and limitations

The major strength of this study is the employment of longitudinal data, allowing for a comprehensive examination of mental health among refugee youth over time. To the best of our knowledge, this study is unique in investigating the trajectories of depression and associated factors among urban refugee youth in LMICs. Findings hold significant implications for understanding the stability and chronicity of mental health problems, particularly depression, among adolescent refugees. They also provide insights into the factors influencing these evolving patterns.

There are also some limitations worth mentioning. First, with n = 164 participants, the study may have been underpowered to accurately identify nuanced trajectories within the data and detect more complex patterns in depression trajectories. Also, by focusing solely on depression as the outcome variable, we may have captured a narrower spectrum of mental health experiences compared to studies that assess composites of psychological problems, potentially limiting the diversity of identified trajectories. Consequently, the interpretations drawn from the study should be approached with caution, and future research with larger sample sizes and multiple outcomes is recommended for validation and extension of the findings. Second, the nonrandom sampling may limit the generalizability of the findings to all urban refugee youth in Kampala, or urban regions in Uganda. Third, the requirement of mobile phone ownership, stemming from the study’s precursor as a digital clinical trial as one of the inclusion criteria, may have excluded the most marginalized urban refugee youth who were affected by the digital divide. This exclusion could have introduced biases and reproduced inequities in research participation. Finally, future research could use more comprehensive measures of violence, such as the whole Conflict Tactics Scale (Straus and Douglas 2004b).

## Conclusion

Changing patterns of mental health over time among refugee youth in LMICs have been largely understudied, due to a lack of longitudinal studies. Our study aimed to fill this gap by investigating the trajectories of depression among urban adolescent and youth refugees in Uganda and assessing the associated socioeconomic and social–ecological factors. We identified a relatively high stability of chronic depression among young urban refugees, underscoring the importance of early screening, prompt risk identification and continuous mental health support interventions. Future studies should consider the intercorrelations between sociodemographic and socioecological factors – such as food insecurity – when investigating the mental health trajectories among refugee populations. Early interventions addressing both mental health support (i.e., coping with traumas, including IPV) and socioeconomic stability (e.g., handling stress due to prolonged stays in informal settlements, life difficulties with young parenthood and food insecurity) are essential for mitigating long-term adverse outcomes among urban refugee youth. Adopting gender-, age- and socioeconomic-tailored approaches to address depression in research, policy and programming can allow for more targeted interventions and supportive strategies with urban refugee youth in LMIC contexts such as Kampala.

## Data Availability

The study data and analysis used in this article can be made available upon reasonable request to the corresponding author.
