# Supplementary material for: Sociodemographic factors associated with trajectories of depression among urban refugee youth in Kampala, Uganda: A longitudinal cohort study
Source: Glob Ment Health (Camb). 2024 Dec 16;11:e125. doi: 10.1017/gmh.2024.135 (PMC11704380; doi:10.1017/gmh.2024.135)
Supplement: Peer review history

# Editor report: Recommendation: Sociodemographic factors associated with trajectories of depression among urban refugee youth in Kampala, Uganda: A longitudinal cohort study — R0/PR2

**DOI:** 10.1017/gmh.2024.135.pr2

**Affiliations:** University of Zimbabwe Faculty of Medicine, Zimbabwe

No accompanying comment.

# Editor report: Decision: Sociodemographic factors associated with trajectories of depression among urban refugee youth in Kampala, Uganda: A longitudinal cohort study — R0/PR3

**DOI:** 10.1017/gmh.2024.135.pr3

**Affiliations:** London School of Hygiene & Tropical Medicine, United Kingdom of Great Britain and Northern Ireland

No accompanying comment.

# Editor report: Recommendation: Sociodemographic factors associated with trajectories of depression among urban refugee youth in Kampala, Uganda: A longitudinal cohort study — R1/PR5

**DOI:** 10.1017/gmh.2024.135.pr5

**Affiliations:** University of Zimbabwe Faculty of Medicine, Zimbabwe

No accompanying comment.

# Editor report: Decision: Sociodemographic factors associated with trajectories of depression among urban refugee youth in Kampala, Uganda: A longitudinal cohort study — R1/PR6

**DOI:** 10.1017/gmh.2024.135.pr6

**Affiliations:** London School of Hygiene & Tropical Medicine, United Kingdom of Great Britain and Northern Ireland

No accompanying comment.
